# A flexible computational pipeline for research analyses of unsolved clinical exome cases

**DOI:** 10.1038/s41525-020-00161-w

**Published:** 2020-12-10

**Authors:** Timo Lassmann, Richard W. Francis, Alexia Weeks, Dave Tang, Sarra E. Jamieson, Stephanie Broley, Hugh J. S. Dawkins, Lauren Dreyer, Jack Goldblatt, Tudor Groza, Benjamin Kamien, Cathy Kiraly-Borri, Fiona McKenzie, Lesley Murphy, Nicholas Pachter, Gargi Pathak, Cathryn Poulton, Amanda Samanek, Rachel Skoss, Jennie Slee, Sharron Townshend, Michelle Ward, Gareth S. Baynam, Jenefer M. Blackwell

**Affiliations:** 1 Telethon Kids Institute, University of Western Australia, Perth, WA Australia; 2 Genetic Services of Western Australia, Department of Health, Government of Western Australia, Perth, WA Australia; 3 Office of Population Health Genomics, Public Health Division, Department of Health, Government of Western Australia, Perth, WA Australia; 4 Faculty of Health and Medical Sciences, Division of Pediatrics, University of Western Australia, Perth, WA Australia; 5 Rare Voices Australia, Sydney, Australia; 6 GaRDN Genetics and Rare Diseases Network, Booragoon, WA Australia; 7 Western Australian Register of Developmental Anomalies, Department of Health, Government of Western Australia, Perth, WA Australia

**Keywords:** Molecular medicine, Genetics research

## Abstract

Exome sequencing has enabled molecular diagnoses for rare disease patients but often with initial diagnostic rates of ~25−30%. Here we develop a robust computational pipeline to rank variants for reassessment of unsolved rare disease patients. A comprehensive web-based patient report is generated in which all deleterious variants can be filtered by gene, variant characteristics, OMIM disease and Phenolyzer scores, and all are annotated with an ACMG classification and links to ClinVar. The pipeline ranked 21/34 previously diagnosed variants as top, with 26 in total ranked ≤7th, 3 ranked ≥13th; 5 failed the pipeline filters. Pathogenic/likely pathogenic variants by ACMG criteria were identified for 22/145 unsolved cases, and a previously undefined candidate disease variant for 27/145. This open access pipeline supports the partnership between clinical and research laboratories to improve the diagnosis of unsolved exomes. It provides a flexible framework for iterative developments to further improve diagnosis.

## Introduction

Exome sequencing (ES) has enabled molecular diagnoses for thousands of rare disease patients (reviewed^1^). Such studies generally report an initial diagnostic rate of ~25−30%^2–8^, generating interest in the development of better computational tools to improve the diagnostic rate. One avenue to achieve this has been through collaboration between clinical genetic services and the research community^9^. For example, Eldomery et al.^10^ recently reported on systematic transfer of molecularly “unsolved” exomes from a clinical to a research setting to accelerate discovery. By recruiting additional family members from 74 initially proband-only ES cases they identified a potential contributing variant in 51% (38/74) of cases. They concluded that additional family members combined with enhanced bioinformatics, including relaxed variant filtering, improves the diagnostic yield. Others also report successful reassessment of unsolved cases leading to improved diagnostic yields, including through enhanced annotation and computational analyses^8,11–13^ as well as through implementation of machine-learning algorithms^14^.

Here we report a study likewise built on the premise of routine transfer of data for unsolved exomes from a clinical service to a research setting to improve diagnosis. A robust, reproducible, and flexible computational pipeline is developed to both aid in diagnosis of unsolved cases and provide a framework for future iterative computational development. The pipeline utilizes open access tools and databases, and incorporates novel scripts and tools developed in-house. Importantly, patient reports include annotation of each variant with American College of Medicine Genetics and Genomics (ACMG)-recommended pathogenicity classifiers^15^ and links to ClinVar^16,17^. In addition to ranking 29/34 prior ES diagnoses used as a reference, candidate variants classified as ACMG pathogenic/likely pathogenic were identified for 22/145 unsolved cases, and a potential novel disease variant for a further 27/145.

## Results

### Participant demographics and clinical indications for genetic diagnosis

Data from 179 consented individuals were suitable for analysis in our pipeline. Of these, 34 (19%) had previously received a molecular diagnosis from GSWA. The research team was initially blinded to the diagnostic laboratory results, which ultimately served as a validation reference for our analysis pipeline. The mean ± SD age of participants at the time of enrolment was 8.03 ± 6.27 years, median age of 6.83, range 0−47 years.

### Summary statistics for variant calling and annotation

One feature that could impact diagnostic accuracy and variant ranking was the variable sequencing technologies employed. Summary statistics are provided in Table 1. Allowing for the difference in capture design, ES using Ion Torrent yielded 1.52 times as many indels per indel-containing gene compared to Illumina, and 0.92 times as many SNVs per gene containing SNVs. These differences are highlighted in Supplementary Fig. 1a where Ion Torrent is seen to yield numerous indels either not present, or at an apparent frequency of 1, in the EXaC_all database compared to fewer such variants in Illumina data. These likely reflect sequence alignment and variant calling errors. Similarly, there are many more missense SNVs called using Ion Torrent compared to Illumina that are absent in EXaC_all (Supplementary Fig. 1b). Caution is therefore required in interpreting variants for molecular diagnoses based on indels and missense variants particularly in Ion Torrent data.

**Table 1:** Summary statistics for variants called using different sequencing technologies.

	Ion Torrent	SOLID	Illumina
*N* samples	56	7	116
Mean ± stdev variants per sample	44,326 ± 6980	35,383 ± 6661	30,985 ± 4935
*N* genes	18637	18527	20270
*N* genes with an indel^a^ (=indelgene)	11,879	5344	9250
*N* genes with an SNV^b^ (=SNVgene)	18,533	18,098	20,120
*N* indels^a^	37,191	7800	19,032
*N* SNVs^b^	209,718	85,859	246,808
Indels^a^/gene	2.00	0.42	0.94
Fold difference (0 = reference)	2.13	0.45	0.00
Indels^a^/indelgene	3.13	1.46	2.06
Fold difference (0 = reference)	1.52	0.71	0.00
SNVs^b^/gene	11.25	4.63	12.18
Fold difference (0 = reference)	0.92	0.38	0.00
SNVs^b^/SNVgene	11.32	4.74	12.27
Fold difference (0 = reference)	0.92	0.39	0.00

^a^Indel = insertion/deletion variant.

^b^SNV = single nucleotide variants.

**Fig. 1:**
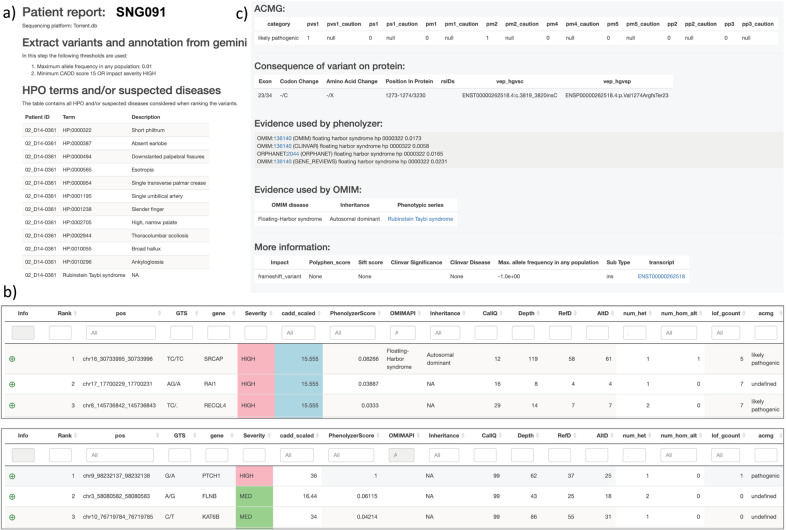
Screenshots for sections of the patient reports output by the diagnostic pipeline. **a** shows the part of the .html file that provides input information including sequencing platform and HPO and OMIM disease terms; **b** shows the top three ranked candidates from two reports where the top candidate ranking is based on OMIM (upper panel) and Phenolyzer score (lower panel). Further columns to the right of the Phenolyzer score provide information from the OMIM API, mode of inheritance, CallQ, total read depth, read depth for the reference allele, read depth for the alternative allele, number of heterozygotes in the cohort, number of homozygotes in the cohort, total number of HIGH impact variants in the given gene across the cohort and ACMG classification. The patient report variant table can be filtered on one or more columns, as desired by the clinician or researcher. Clicking on the green dot to the left of the rank 1 variant in the upper panel shows c the evidence used to diagnose this particular variant as Floating Harbor Syndrome based on the OMIM Phenotypic Series for Rubinstein Taybi Syndrome.

### Pipeline performance for previously diagnosed cases

The pipeline ranked 21/34 previous diagnoses as the top candidate, with 26 in total ranked ≤7th, and 3 ranked ≥13th (Table 2 and Supplementary Data 1). One variant was undetected due to being on the uncaptured mitochondrial genome, four failed filtering parameters, 2 with CallQ < 10, 2 with VQSLOD > 99.7. The latter two variants (*KMT2D* and *TRPS1*) received a ranking of 1 when this cut-off was relaxed. Of the 29 subjects achieving a diagnosis in the pipeline, 12 were sequenced on Ion Torrent, 15 on Illumina TruSight™, and 2 on SOLiD (Table 2). Impact severity (Table 2) was HIGH (frameshift; stop-gain; splice-site) for 7/15 Illumina, 5/12 Ion Torrent and 1/2 SOLiD diagnoses, and MEDIUM (missense) for 8/15 Illumina, 7/12 Ion Torrent and 1/2 SOLiD diagnoses. Diagnosis was achieved directly through the OMIM API for 22 exomes (two through the phenotypic series), 11 of which also returned a Phenolyzer score of 1 (Table 2). Diagnosis was achieved in five cases using Phenolyzer scores alone. The average Phenolyzer score for OMIM-diagnosed cases was (mean ± SD) 0.63 ± 0.40 compared to 0.16 ± 0.37 for diagnoses made using only Phenolyzer scores. Mode of inheritance was autosomal dominant for 17, autosomal recessive for 7, X-linked dominant for 1, and unknown for 5 cases. A total of 21 diagnoses were classified as pathogenic/likely pathogenic using ACMG classifiers, 9 of which were not present in ClinVar. Eight diagnoses were undefined. Of the cases where our pipeline did not perform well (i.e. variants ranked ≥13) we observe (Table 2): (i) the diagnosed variant ranked 13 was a hemizygous *PTCHD1* c.2489 T > G (p.Ile830Arg) variant that received a Phenolyzer score of 0.027 (i.e. 12 other gene variants were ranked higher by Phenolyzer based on HPO terms); (ii) for the diagnosed variant ranked 19, the clinical laboratory diagnosis was based on compound heterozygosity at *SKIV2L* c.904 C > T (p.Gln302*) and c.2662_2663delAG (p.Arg888Glyfs*12), only the first of which was present in the SOLiD data with a low Phenolyzer score (0.001; 18 other variants gave higher Phenolyzer scores); and (iii) for the diagnosis with variants ranked 27 and 37, the clinical diagnosis was based on compound heterozygosity at *SARDH* (c.1442 G > A p.(Arg481His) and c.2032 G > A p.(Glu678Lys)) with neither variant identified by Phenolyzer. More recent review of this patient by RUDDS^9^ suggests that these variants may be benign (G.S.B.). None of these lower ranked variants were identifiable through the OMIM API, and all were classed as “undefined” using our ACMG classifier.

**Table 2:** Details of pipeline performance for the 34 previously diagnosed cases.

Patient ID	Rank	Gene	Zygosity	OMIM API?	Phenotypic series	Phenolyzer score	ACMG criteria	ACMG	ClinVar	Reported MoI	Type of variant	Variant^*a*^	rs ID	*N* HPO terms	*N* Dis Terms	N genes with rare variant	CallQ	Depth	Ref	Alt	LoF	*N* Het	*N* Hom Alt
Illumina																							
SNG038	1	*WT1*	Het	1	1	0.782	pvs1,pm1,pm2	P	None	AD	frameshift	p.Leu99Ter	None	11	2	101	99	45	26	19	1	1	0
SNG081	1	*TBX5*	Het	1	0	1.000	pvs1,pm1,pm2,pp3	P	P	AD	missense	p.Thr223Met	None	5	1	134	99	36	24	12	0	1	0
SNG114	1 2	*SLC26A4*	Het Het	1	0	0.740	pvs1,ps1,pm1,pm2,pp3 pm2,pp3	P VUS	P VUS	AR	missense missense	p.Arg409His p.Glu29Gly	rs111033305 rs1446406563	5	1	108	99 99	61 28	27 15	34 13	0	1 1	0 0
SNG161	4	*ARX*	Het	0	0	0.062	pvs1pm1pm2	P	None	XL/XLR	frameshift	p.Glu541GlyfsTer132	None	9	0	130	96	32	0	32	1	0	1
SNG023	1	*ASPM b*	Het	1	0	0.717	pvs1,pm1,pm2	P	None	AR	stop-gain	p.Arg577Ter	rs886039310	9	1	125	99	101	56	45	2	2	0
SNG172	1	*COL7A1*	Het	1	0	1.000	pvs1,ps1,pm1,pm2	P	P	AD	missense	p.Gly2032Arg	rs866061439	0	1	182	99	130	63	67	0	1	0
SNG130	1	*IRF6*	Het	1	0	1.000	pvs1,ps1,pm2	P	P	AD	stop-gain	p.Trp192Ter	rs886039389	4	1	105	99	151	84	67	1	1	0
SNG175	1	*RASA1*	Het	0	0	1.000	pvs1,pm1,pm2	P	None	AD	stop-gain	p.Tyr376Ter	None	19	2	115	99	44	24	20	1	1	0
SNG173	1	*TEK*	Het	1	0	1.000	pvs1,ps1,pm1,pm2	P	P	AD	missense	p.Tyr749Ser	rs80338909	2	2	128	99	73	37	36	0	1	0
SNG022	1	*EFNB1*	Het	1	0	0.001	ps1,pm1,pp3	LP	LP	XLD	missense	p.Pro54Leu	rs104894801	4	1	85	99	115	56	59	0	1	0
SNG064	1	*SOX10*	Het	1	0	1.000	pvs1,pm2	LP	None	AD	frameshift	p.Glu359ArgfsTer143	None	4	1	119	99	53	25	28	1	1	0
SNG035	1	*TRPS1*	Het	1	0	1.000	pvs1,pm2	LP	None	AD	frameshift	p.Tyr1144LeufsTer18	None	19	1	115	99	180	84	96	1	1	0
SNG200	27 37	*SARDH*	Het Het	0	0	0.000	pp3 pm1,pm2,pp3	VUS VUS	None None	AR	missense missense	p.Arg481His p.Glu678Lys	rs35699831 rs142376496	39	1	126	99 99	104 119	39 54	65 65	1	1 1	0 0
SNG199	13	*PTCHD1*	Hom	0	0	0.027	pm1,pm2,pp3	VUS	None	XLR	missense	p.Ile830Arg	None	13	0	116	99	45	0	45	0	0	1
SNG095	1	*DNMT3*	Het	1	0	1.000	pm1,pm2,pp3	VUS	None	AD	missense	p.Ile634Thr	rs1390273539	17	1	100	99	90	46	44	0	1	0
SNG059	0	***RASA1***	CallQ < 10											11	2	107							
SNG093	0	***FOXG1***	CallQ < 10											9	0	95							
SNG145	0	***KMT2D***	VQSLOD > 99.7											25	1	47							
SNG148	0	***MT-ND4***	Mitochondrial genome											18	1	126							
SNG174	0	***TRPS1***	VQSLOD > 99.7											2	2	35							
Mean				0.73	0.07	0.689								10.67	1.07	119.27	98.79	82.57	39.43	43.14			
SD				0.46	0.26	0.428								9.93	0.59	21.84	0.80	46.69	26.68	22.82			
Ion Torrent																							
SNG004	1	*PIK3CA*	Het	1	0	0.003	pvs1,ps1,pm1,pm2	P	P	n/k	missense	p.Cys378Tyr	rs397514565	2	1	386	30	44	32	12	1	1	0
SNG019	1	*RIT1*	Het	1	0	0.280	pvs1,ps1,pm1,pm2,pp3	P	P	AD	missense	p.Gly95Ala	rs672601335	0	1	921	99	97	42	55	0	1	0
SNG021	1	*CREBBP*	Het	1	0	0.164	pvs1,pm1,pm2	P	None	AD	stop-gain	p.Arg1498Ter	None	9	1	765	65	30	12	18	1	1	0
SNG027	3	*MAP2K1*	Het	1	1	0.004	pvs1,ps1,pm1,pm2,pp3	P	P	AD	missense	p.Tyr130Cys	rs121908595	12	0	666	108	69	29	40	0	1	0
SNG028	1	*COL7A1*	Het	1	0	1.000	pvs1,ps1,pm1,pm2	P	P	AD	frameshift	p.Gly1281ValfsTer44	rs757688782	3	1	476	70	129	68	61	7	1	0
SNG062	2	*LAMB3*	Hom	1	0	0.308	pvs1,pm1,pm2	P	None	AR	stop-gain	p.Gln73Ter	rs762234799	0	1	807	32	70	0	70	3	0	1
SNG105	1	*BRAF*	Het	1	0	1.000	pvs1,ps1,pm1,pm2,pp3	P	P	AD	missense	p.Thr207Ile	rs121913375	16	1	845	99	138	71	67	2	1	0
SNG106	1	*KRAS*	Het	1	0	0.181	pvs1,ps1,pm1,pm2,pp3	P	P	AD	missense	p.Phe156Ile	rs397517042	10	1	580	21	10	6	4	0	1	0
SNG066	1	*TCF4*	Het	1	0	1.000	pvs1,pm2	LP	None	AD	frameshift	p.Leu319ThrfsTer8	None	14	1	718	99	89	42	47	2	1	0
SNG012	1	*UBE3A*	Het	1	0	0.431	pm2	VUS	None	AD	splice acceptor	c.2508-1G>A;NA	None	14	1	921	99	74	38	36	1	1	0
SNG017	4	*LOXc*	Hom	0	0	0.029	pm1,pm2,pp3	VUS	None	AR	missense	p.Thr341Pro	None	3	0	839	23	51	0	51	0	0	2
SNG018	7	*LOXc*	Hom	0	0	0.029	pm1,pm2,pp3	VUS	None	AR	missense	p.Thr341Pro	None	3	0	465	37	83	0	83	0	0	2
Mean				0.83	0.08	0.369								7.17	0.75	699.08	65.17	73.67	28.33	45.33			
SD				0.39	0.29	0.403								5.94	0.45	184.28	34.77	37.49	25.31	24.41			
SOLiD																							
SNG008	1	NRAS	Het	1	0	0.235	pm1,pm2,pp3	VUS	None	AD	missense	p.Thr58Ile	None	7	1	255	38	10	5	5	0	1	0
SNG045	19	SKIV2L	Het	0	0	0.001	pm2	VUS	None	AR	stop-gain	p.Gln302Ter	rs751074844	22	0	291	100	175	116	59	1	1	0

Bold highlights the previously diagnosed cases that failed in the pipeline, reasons for which are annotated onto the table. Rank indicates rank in our pipeline; OMIM API? indicates whether the diagnosis was made by direct access to the OMIM API (1 = yes; 0 = No); Phenotypic Series indicates whether the diagnosed gene was identified via the phenotypic series in OMIM (1 = yes; 0 = No). *N* Het is the number of individuals in the cohort heterozygous for the variant; *N* Hom the number of homozygous individuals. Two patients were diagnosed as compound heterozygotes.

*P* pathogenic, *LP* likely pathogenic, *VUS* variant of unknown significance (categorized as undefined in our pipeline), *LoF* number of loss-of-function variants seen in this gene in the cohort.

^a^Full details of transcripts are provided in Supplementary Table S2.

^b^Compound heterozygote, second variant did not filter through the pipeline

^c^Same variant related individuals.

We compared rankings for the 29 subjects with a previous diagnosis between our pipeline and those achieved using Exomiser^18^ and AMELIE^19^ (Table 3). Compared to our pipeline, AMELIE achieved a better rank in 14%, the same rank in 34%, and a lower rank in 52% of cases. Similarly, Exomiser achieved a better rank in 7%, the same rank in 41%, and a lower rank in 52% of cases.

**Table 3:** Rankings obtained for 29 previously diagnosed cases using our SeqNextGen pipeline compared to Exomiser^18^ and AMELIE^19^.

Patient ID	Sequencing platform	SeqNextGen	AMELIE	Exomiser
SNG038	Illumina	1	3	1
SNG081	Illumina	1	1	1
SNG114	Illumina	1	1	1
SNG161	Illumina	4	4	1
SNG024	Illumina	1	13	0
SNG172	Illumina	1	0^a^	0^a^
SNG130	Illumina	1	1	1
SNG175	Illumina	1	25	9
SNG173	Illumina	1	2	1
SNG022	Illumina	1	9	1
SNG064	Illumina	1	1	1
SNG035	Illumina	1	3	1
SNG200	Illumina	27	31	45
SNG199	Illumina	13	4	3
SNG095	Illumina	1	4	1
SNG005	Ion Torrent	1	8	2
SNG019	Ion Torrent	1	1	2
SNG021	Ion Torrent	1	1	4
SNG027	Ion Torrent	3	17	15
SNG028	Ion Torrent	1	1	1
SNG062	Ion Torrent	2	0	0
SNG105	Ion Torrent	1	13	3
SNG106	Ion Torrent	1	1	1
SNG066	Ion Torrent	1	2	0
SNG012	Ion Torrent	1	19	10
SNG017	Ion Torrent	4	3	205
SNG018	Ion Torrent	7	5	120
SNG008	Solid	1	1	1
SNG045	Solid	19	14	84
		Better rank (%)	4 (14%)	2 (7%)
		Same rank (%)	10 (34%)	12 (41%)
		Worse rank (%)	15 (52%)	15 (52%)
		Total	29	29

^a^AMELIE and Exomiser were unable to give a ranking for this subject as no HPO terms were available. The SeqNextGen pipeline used OMIM terms.

### Pipeline performance for previously unsolved cases

As noted previously^12^, reviewing putative diagnostic variants from ES data is challenging. Based on the performance of our pipeline in the reassessment of previously diagnosed cases, we focused our initial review of putative candidate variants on those ranked ˂10. This was carried out by initial manual review of the patient reports for the 145 unsolved cases by a member of the research team who only had access to HPO terms for phenotypic information. This was followed by a review of candidate diagnoses by at least two members of the clinical team who then had full access to the patient’s full clinical notes and history. Screenshots for sections of example output files show the part of the report that provides input information including sequencing platform and HPO and OMIM disease terms (Fig. 1a) and the top three ranked candidates from two reports (Fig. 1b) where the top candidate ranking is based on OMIM (upper panel) or Phenolyzer score (lower panel). Columns to the right of the Phenolyzer score provide information from the OMIM API, mode of inheritance, CallQ, total read depth, read depth for the reference allele, read depth for the alternative allele, number of heterozygotes in the cohort, number of homozygotes (for the alternative allele) in the cohort, total number of HIGH impact variants in the given gene across the cohort, and ACMG classification. The patient report variant table can be filtered on one or more columns, as desired. Clicking on the green dot to the left of rank 1 gene in Fig. 1b (upper panel) shows the evidence (Fig. 1c) used to diagnose the variant as Floating Harbor Syndrome based on the OMIM Phenotypic Series for Rubinstein Taybi Syndrome. For this patient the suspected clinical syndrome Rubinstein Taybi was indicated by the attending clinical geneticist (Fig. 1a). Detailed evidence used by the ACMG classifier to determine variant pathogenicity is also shown in this dropdown panel (Fig. 1c). Examples of the patient reports that can be viewed in a web-browser are available at https://richardwfrancis.github.io/sng_reports/.

Based on the review of the 145 patient reports for unsolved cases (Supplementary Data 1), a candidate variant classified as pathogenic/likely pathogenic by ACMG criteria was identified for 22/145 previously unsolved cases, 13 of which were not present in ClinVar. The remaining nine were categorized as pathogenic/likely pathogenic in ClinVar. A potential novel candidate variant (i.e. classified “undefined”) was identified for a further 27/145 (Supplementary Data 1). Of these 49 putative variants, 18/145 were deemed of immediate clinical relevance (Table 4); others remain under clinical review. For the 49 putative candidate variants, 48 were ranked ≤7th (Supplementary Data 1). Except in the case of one putative compound heterozygote, none of these candidate diagnoses were heterozygous for variants previously associated with autosomal recessive disorders. Candidate variants were based on the OMIM API for 13/49 (27%) subjects, compared to prior diagnoses where 76% (22/29) were based on access to this resource. Mirroring this, Phenolyzer scores were also generally lower for candidate variants compared to prior diagnosed variants (Tables 2 and 4). It should be noted, however, that Phenolyzer scores depend on the number and relative ranking of candidate genes within an individual and are not normalized across individuals. There were no significant differences in mean ± SD read depth (Supplementary Data 1) for Ion Torrent (prior diagnoses: 73.67 ± 37.49; candidate diagnoses: 80.85 ± 58.17) compared to Illumina TruSight™ (prior: 84.23 ± 48.16; candidate: 70.26 ± 58.17) sequence data for either previous or candidate diagnosed cases. Mean CallQ for variants called using Ion Torrent (65.17 ± 34.77) was significantly (*p* = 0.007) lower than for Illumina (98.77 ± 0.83) for prior diagnoses, with a similar trend for candidate diagnoses (*p* = 0.15; Ion Torrent: 69.65 ± 73.43; Illumina 94.30 ± 15.08). Most candidate variants classified as pathogenic/likely pathogenic by ACMG criteria were HIGH impact variants, all of which were frameshifts for Ion Torrent whereas Illumina diagnoses were mostly stop-gain or start-loss (Supplementary Data 1). A number of variants were observed >1 in our cohort (as were two of the prior diagnosed variants, Table 2). Given the potential for sequence alignment errors, we only retained repeat candidate variants where the variant was classified ACMG pathogenic/likely pathogenic (or once for a possible compound heterozygote) and the frequency of the variant in our cohort was 0.006 (2/358 chromosomes), i.e. below the accepted cut-off of 0.01 for rare alleles in the population. Information on ranked variants replicated more than twice in our cohort were retained under a list of unresolved variants for review by the clinical genetics team (Supplementary Data 1). For both sequencing technologies the majority of putative novel (i.e. not previously associated with a rare disease phenotype) variants were MEDIUM impact missense variants, reinforcing the imperative for functional data to support validation of these variants.

**Table 4:** Details of pipeline performance for 18 candidate molecular diagnoses for previously unsolved cases based on Illumina TruSight or Ion Torrent sequencing as indicated. These 18 candidate variants were considered of immediate clinical relevance upon clinical review.

Patient ID	Rank	Gene	Zygosity	OMIM API?	Phenotypic Series	Phenolyzer score	ACMG criteria	ACMG	ClinVar	Reported MoI	Type of variant	Variant^*a*^	rs ID	*N* HPO terms	*N* Dis Terms	*N* genes with rare variant	CallQ	Depth	Ref	Alt	Lof	*N* Het	*N* Hom
Illumina																							
SNG190	1	*PTCH1*	Het	0	0	1.000	pvs1,ps1,pm1,pm2	P	P	AD	stop-gain	p.Arg267Ter	rs863224650	8	0	145	99	62	37	25	1	1	0
SNG217	1	*EHMT1*	Het	0	0	0.764	pvs1,ps1,pm1,pm2	P	P	AD	stop-gain	p.Arg1168Ter	rs121918301	27	0	218	99	130	71	59	2	1	0
SNG178	2	*ARID1A*	Het	0	0	0.127	pvs1,pm1,pm2	P	None	AD	frameshift	p.Val1024AlafsTer10	None	14	0	207	99	143	120	12	1	1	0
SNG129	5	*NFIX*	Het	0	0	0.038	pvs1,pm1,pm2	P	None	AD	stop-gain	p.Gln232Ter	None	25	4	100	99	70	35	35	1	1	0
SNG133	7	*GLI2*	Het	0	0	0.003	pvs1,pm2	LP	None	AD	frameshift	p.Ala641ProfsTer59	None	9	1	138	78	4	2	2	1	1	0
SNG057	3	*ANKRD11*	Het	0	0	0.026	pm2	Undefined	None	AD	stop-gain	p.Ser1884Ter	None	16	1	108	99	49	23	26	1	1	0
SNG152	4	*GAS1*	Het	0	0	0.002	pm2,pp3	Undefined	None	AD	missense	p.Arg45Gly	rs922820794	4	0	118	31	6	2	4	0	1	0
SNG212	1	*LRP5*	Het	0	0	0.161	pm2,pp3	Undefined	None	AD	missense	p.Thr297Ile	rs765013945	11	0	233	99	96	36	60	0	1	0
SNG197	4	*BMP2*	Het	0	0	0.058	pm1,pp3	Undefined	None	AD	missense	p.Arg131Ser	rs140417301	13	0	109	99	47	26	21	0	1	0
SNG058	1	*EHMT1*	Het	1	0	0.044	pm2	Undefined	None	AD	splice-donor	c.3716+1G>T	None	15	1	99	99	34	17	17	2	1	0
SNG086	1	*SHANK3*	Het	0	0	0.261	pp3	Undefined	VUS	AD	missense	p.Pro1665Thr	rs749130556	15	0	111	99	35	14	21	0	1	0
Mean				0.09	0.00	0.23								14.27	0.64	144.18	90.91	61.45	34.82	25.64			
SD				0.30	0.00	0.34								6.83	1.21	50.65	20.84	45.62	34.20	19.27			
Ion Torrent																							
SNG113	1	*KMT2D*	Het	1	1	0.000	pvs1,pm2	LP	None	AD	missense	p.Gly965Arg	None	13	1	830	20	15	5	10	22	1	0
SNG107	4	*FRMPD4*	Hom	1	1	0	pm2	Undefined	None	XLR	missense	p.Gly691Val	rs200183778	11	1	845	200	45	45	0	0	0	1
SNG025	1	*RAI1*	Het	0	0	0.392	pm2	Undefined	None	AD,IC	frameshift	p.Asn1254LysfsTer61	None	17	0	805	28	91	69	22	7	1	0
SNG078	1	*TCF4*	Het	0	0	0.366	pm2,pp3	Undefined	None	AD	missense	p.Ser78Cys	rs780638244	12	0	398	99	86	35	51	2	1	0
SNG084	1	*RAI1*	Het	1	1	0.796	pm2	Undefined	None	AD	missense	p.Gln306Ter	rs61753380	17	1	805	99	128	64	64	7	1	0
SNG060	1 2	*EPG5*	Het Het	1	0	1	pm2 pm2,pp3	Undefined Undefined	None None	AR	splice variant missense	c.6049+5G>A p.Ile1185Leu	None None	17	1	525	99 99	174 124	92 59	82 59	0	1 1	0 0
SNG112	1	*BRAF*	Het	0	0	0.773	pm2	Undefined	None	AD	frameshift	p.Gly30AlafsTer24	None	16	0	412	15	23	9	14	2	1	0
Mean				0.57	0.43	0.48								14.71	0.57	660.00	76.83	64.67	37.83	26.83			
SD				0.53	0.53	0.40								2.63	0.53	205.57	71.54	44.16	26.93	25.13			

Three (SNG190, SNG178, SNG057) have been validated by Sanger sequencing of patient plus parents. Of these 18 variants, 6 classified as ACMG pathogenic or likely pathogenic, the remainder are undefined. Full details of these variants are provided in Table S2. Details of a further 27 putative candidate diagnoses that remain under clinical review are provided in Table S2. Rank indicates rank in our pipeline; OMIM API? indicates whether the diagnosis was made by direct access to the OMIM API (1 = yes; 0 = No); Phenotypic Series indicates whether the diagnosed gene was identified via the phenotypic series in OMIM (1 = yes; 0 = No). *N* Het is the number of individuals in the cohort heterozygous for the variant; *N* Hom the number of homozygous individuals. One patient was listed as a possible compound heterozygote.

*AD* autosomal dominant, *AR* autosomal recessive, *IC* isolated cases, *P* pathogenic, *LP* likely pathogenic, *VUS* variant of unknown significance (categorized as undefined in our pipeline), *LoF* number of loss-of-function variants seen in this gene in the cohort.

^a^Full details of transcripts are provided in Table S2.

### Features of unresolved unsolved cases

It was not possible to assign candidate pathogenic variants for 96 unresolved cases through our pipeline (Supplementary Data 1). Where a feasible candidate was ranked by OMIM or Phenolyzer scores, we retained the information in the table for clinical review. This included instances where the phenotype was correct for the gene but the mode of inheritance was wrong, including instances where ≥3 individuals carried the variant in our cohort. There were 44 patients for whom there were no ranked candidates based on OMIM or Phenolyzer scores. Further features of the sequence data for these unresolved cases are provided in the footnote to Supplementary Data 1. Failure in the pipeline did not appear due to sequencing technology per se, or the number of HPO terms available. Possible explanations for our failure to identify a candidate pathogenic variant include the gene not being in the capture panel employed, the variant being captured but pathogenicity not assigned to it, either because it was not possible to assign definitive ACMG criteria and/or the relationship between this gene and the clinical phenotype not yet being reported in public domain databases. As the knowledge in public domain databases expands, reanalysis of the current data could lead to future identification of a disease-causing variant.

## Discussion

Here we developed and implemented a computational pipeline to reassess exome data from previously unsolved cases as a research partnership with clinical services. Our primary purpose here is to report on the potential for this computational pipeline to rank variants using a variety of tools to capture both phenotypic input and variant evaluation according to ACMG guidelines and ClinVar entries. The primary research output is the comprehensive, intuitive web-based report (html file) generated for each patient which can readily be reviewed on a case-by-case basis and provides a summary for all putative deleterious variants in the individual. We undertook a review of unresolved cases compared to previously diagnosed cases to determine the potential of our pipeline to identify and rank further candidate pathogenic variants. The 49 candidate variants identified in this research setting should not be viewed as diagnostic variants per se but are selected to guide the team of clinical geneticists to review cases in the cohort. Although most are not yet validated diagnostic variants, on clinical review 18/49 were considered of immediate relevance and have been taken forward for clinical diagnosis; three have been validated by Sanger sequencing of patient plus parents.

Evaluation of different sequencing technologies was not a primary focus of our study. Nevertheless we, like others^20,21^, found that it was important to be aware of differences in rates of systematic sequencing and alignment errors in generating a ranked list of candidate variants. In its current form the pipeline benefits from accessing the OMIM API to match for genes associated with specific disease terms, or in a phenotypic series, as well as interrogating disease and HPO terms in Phenolyzer^22^. Although the Phenolyzer^22^ tool itself accesses public domain gene−disease databases (OMIM, Orphanet, ClinVar, Gene Reviews, and GWAS catalogue), we found that direct interrogation of the OMIM API could provide a candidate molecular diagnosis when Phenolyzer failed or gave a very low score. This is assisted by incorporation of the OMIM phenotypic series, which is not currently implemented in Phenolyzer, and by more up-to-date information in OMIM compared to the incidence of OMIM stored within Phenolyzer. Nevertheless, the simple implementation of these two resources has been successful in (a) identifying a high proportion (29/34) of previously diagnosed variants; (b) providing a good yield (22/145) of candidate molecular variants for unsolved cases classified as ACMG pathogenic/likely pathogenic; and (c) providing a potential novel disease variant for a further 27/145 unsolved cases. While the latter will provide more of a challenge for validation, we note that not all the previously diagnosed variants were classified as ACMG pathogenic/likely pathogenic. In addition, 9/21 (43%) diagnosed variants classified as ACMG pathogenic/likely pathogenic, and 13/22 (59%) candidate variants classified as ACMG pathogenic/likely pathogenic, were not present in ClinVar^17^. ClinVar directly accepts rather than curates classifications from submitters. Here we took a systematic approach to assign ACMG classification based on existing evidence in the public domain. While there was significant overlap in classification between our method and results found in ClinVar, it is not surprising that differences occur. Furthermore, as there are no strict rules on implementing ACMG guidelines in terms of which tools, resources and methods to use, even other systematic approaches may yield differing clinical significance classifications for the same variant. Overall, our pipeline to re-evaluate clinical sequence data for unsolved exomes contributes to the growing number of reports^4,8,10–14^ demonstrating that such reassessment can improve the diagnosis of rare diseases.

Although the initial focus in this partnership was to reassess unsolved cases, our research aim was to build a robust but flexible pipeline that would provide a framework for future iterative computational development. While the pipeline ranks genes most relevant to a patient’s clinical phenotype, the patient report provides information on all putative deleterious variants which the researcher and/or clinical team can sort and re-rank based on all classifiers. As also found by others^23–26^, the use of HPO terms provides important input to our pipeline. The HPO was developed to provide a consistent and standardized vocabulary of phenotypic abnormalities that result from genetic disorders and is currently the most complete vocabulary used in the rare disease field. The Clinical Genetics Service partner in this study has now implemented the tool PatientArchive^27^ (https://mme.australiangenomics.org.au/#/home), a clinical grade phenotype-oriented patient data management platform that allows clinicians to use free text clinical notes for structured patient phenotyping that are automatically translated into HPO terms. The platform enables patient data management, collaborative diagnosis and knowledge exchange within Australia, and is also part of the global GA4GH MatchMaker Exchange Initiative^27^ (http://www.matchmakerexchange.org/). Automated input of HPO and disease term data from PatientArchive into our computational pipeline has streamlined the transfer of data from the clinical to the research diagnostic setting. However, the fact that not all candidates identified in the research setting, where the research team only had access to HPO terms as phenotypic indicators, were immediately obvious as candidates following clinical review indicates that the translation of clinical information to standardized HPO terminology is not yet perfect. Iterative improvements in clinical reporting and the ability of PatientArchive to identify the best set of HPO terms to describe the patient’s clinical phenotype will be important in improving the accuracy of our pipeline in ranking variants for clinical review. Others have also recently published on how to choose an optimal set of HPO terms and enter them using PatientArchive^26^. Incorporating a step in the pipeline which allows us to classify all variants according to ACMG criteria for pathogenic/likely pathogenic status also provides an important aid for feedback of research candidate variants to the clinical diagnostic team. At present we have not used ACMG criteria in the prioritization of variants since we don’t wish to compromise the potential for identification of novel candidate variants based on phenotype. However, in developing their X-rare machine-learning method for rare disease diagnosis, we note that Li et al.^14^ recently devised a weighted sum ACMG score based on the 14 implemented evidences proposed by ACMG to represent the overall pathogenic/benign strength. This Xrare_ACMG score performed better than other computational genotype-only scores. Others have also reported^28^ on semi-automated methods for implementing ACMG criteria within the tool InterVar. Such tools could be further evaluated and implemented within our flexible pipeline. Implementation of a greater range of variant prioritization tools within the pipeline, taking account of phenotype-specific differences in performance, could also enhance our ability to rank variants compared to our current use of scaled CADD scores alone. For example, we recently carried out a phenotype centric benchmarking of a range of variant prioritization tools (including best performers FATHMM, M-CAP, MetaLR, MetaSVM and VEST3), demonstrating that the performance of these tools varies according to disease context^29^. Current research in the laboratory also focuses on the incorporation of public domain data^30–33^ on tissue- and cell-specific gene expression to improve predictive algorithms. Additional routines could be implemented to address copy number variation and chromosomal anomalies, although all patients included in this study had prior chromosomal microarray analysis. Finally, our research partnership with clinical genetic services is now undertaking analysis of genome sequencing (GS) which is driving further development of the pipeline. Lionel et al.^34^ recently demonstrated improved diagnostic yield using GS compared to targeted gene sequencing panels and ES, due both to improved exonic coverage as well as to structural and non-exonic sequence variants not detectable with ES.

In summary, an accurate diagnosis informs prognosis and can positively impact on management for individuals living with a rare disease and their families. We have developed a robust computational pipeline that is automated, is built in a framework that can incorporate novel tools and public domain data as they become available, improving the accuracy of molecular diagnoses for rare diseases. Our pipeline supports the principle^10^ that systematic transfer of molecularly “unsolved” exomes from a clinical to a research setting will accelerate human genetic disease discovery.

## Methods

### Study design and participants

Ethical approval for the study (known as SeqNextGen) was obtained from the Human Research Ethics Committee at Princess Margaret Hospital for Children, Perth, Australia (#2105034EP) and the Department of Health Research Governance Service (#RGS2494). Participants were recruited through a genetic counsellor at Genetic Services of Western Australia (GSWA), King Edward Memorial Hospital, Perth, Australia. All individuals were engaged through the RUDDS^9^. Participants, or their carers (for participants aged <18 years of age or >18 years with reduced capacity to consent), gave written consent to share their de-identified ES or targeted NGS-sequenced exome data and relevant clinical phenotypic information with the SeqNextGen study. Only a clinical geneticist at Genetic Services of Western Australia had the authority to re‐identify a participant to provide feedback of genetic results as they pertained to the rare disease diagnosis. Feedback is only provided for fully validated variants. Secondary findings (i.e. information on genetic variants not related to the individual’s primary rare disease phenotype) were not gathered or reported. Participants were eligible to take part in the SeqNextGen study if they had given prior clinical consent for genetic diagnosis of their rare disease using ES/targeted exome NGS. All phenotypes, except for neuromuscular disease phenotypes (which in RUDDs are channelled through an alternative diagnostic pathway), were eligible for inclusion in the study; there were no exclusion criteria.

### Sequencing data and variant detection

ES/targeted exome NGS was carried out by a diagnostic genomics laboratory. Our cohort was sequenced under different protocols and sequencing platforms due to technological advances and changes in the diagnostic service through time. The TargetSeq Exome V2 kit was used for exome enrichment for sequencing on the SOLiD system. SOLiD ES data were analysed under LifeScope 2.5, with default parameters for exome analysis. The Ion AmpliSeq Exome RDY kit was used for exome enrichment for sequencing on the Ion Proton System. Ion Torrent ES data were analysed under Torrent Suite 4.2, using Thermo Fisher’s default exome-customized analysis parameters. Illumina ES analysis was carried out using TruSight™ One (~4800 genes) or TruSight™ One Expanded (~6700 genes) panels (Illumina Inc., Victoria, Australia) sequenced on the MiSeq or NextSeq 550 systems and analysed using MiSeq Reporter. Human genome version 19 (hg19) was used as the reference genome in all cases.

### Storage and processing of variants in GEMINI

Variant call format (VCF) files for Ion Torrent and SOLiD sequencing data were as provided by the service laboratory. For Illumina data, BAM files were processed with GATK 4.0.2.0 ^35,36^ and SAMtools 1.7 ^37^ using an ‘intersect-then-combine’ approach. Variant calling was performed with GATK following best practices^38^ using 99.7 as the truth sensitivity threshold at the ApplyVQSR stage of the pipeline (https://software.broadinstitute.org/gatk/best-practices/workflow?id=11145) and with SAMtools^37^ using the mpileup function. Only variants identified by both methods were retained.

Patient data were grouped and processed based on sequencing technology used. Variants were decomposed and normalized using vt^39^ (version v0.57721) to ensure all variants were represented in a unified manner regardless of variant calling software. Variants were annotated using the Ensembl Variant Effect Predictor (VEP) version 84^40^. This includes predicted deleteriousness scores from SIFT^41^, PolyPhen2^42^, and CADD^43^, allele frequencies from ExAC^44^, the 1000 Genomes Project (1KGP)^45^, and NHLBI GO Exome Sequencing Project (ESP)^46^. The annotation also provides HUGO Gene Nomenclature Committee gene symbols, variant information with respect to transcripts and proteins including Human Genome Variation Society (HGVS) expressions, and functional consequences using Sequence Ontology (SO) terms. The resulting annotated variants were imported into GEMINI^47^ (version 0.30.1), a flexible system for storing and querying genetic variants, along with metadata from the VCF file, such as coverage depth and zygosity of a variant. The only categories of variants analysed here were insertion/deletion (indels) and single nucleotide variants (SNVs). Impact severity for variants was classified based on SO terms HIGH, MEDIUM or LOW as used within GEMINI.

### Parameters for variant filtering

Variants with a call quality (CallQ) ˂ 10 were discarded as were synonymous variants and intron variants, identified by the GEMINI SO terms “synonymous_variant” and “intron_variant”, respectively. Two thresholds were used to further filter variants with call quality of ≥10: (1) maximum allele frequency in any population (ExAC_all, 1KGP, and ESP databases) of 0.01; and (2) minimum scaled CADD score 15 OR impact severity HIGH.

### Using the OMIM API to match disease terms with genes

OMIM disease terms related to potential diagnoses were available for 95/179 (53%) of the patients. We developed a tool, Phenoparser, which uses the OMIM API to match a given term to OMIM phenotypes and return any associated genes and their synonyms/aliases. The search capability within the OMIM API is powered by an open-source enterprise search platform called Apache Lucene Solr, which provides essential search features, such as spellcheck and thesaurus matching^48^. Specifically, a “text search” is performed to match the available OMIM terms to all fields of an OMIM entry except external data fields. Within the results, only those where a gene map is available (gm_phenotype_exists is true) are returned along with gene-to-disease associations from OMIM. Phenoparser also retrieves similar data for any available Phenotypic Series records—a collection of entries with overlapping clinical manifestations. Phenoparser queries the online database and thereby always retrieves the most up-to-date information at the time of running. However, the results obtained from the specific incidence of the OMIM API interrogated on any given date is stored within an SQLite database as a cross-reference for any future reanalysis of an individual patient’s exome data.

### Using Phenolyzer to match HPO terms with genes

Patient clinical phenotypes were converted into HPO terms^49,50^ using the tool Patient Archive^27^ (https://mme.australiangenomics.org.au/#/home), a clinical grade phenotype-oriented patient data management platform that allows clinicians to use free text clinical notes for structured patient phenotyping that are automatically translated into HPO terms. HPO terms for 176/179 (98%) patients were combined with available OMIM terms and the phenotype-based gene analyser tool, Phenolyzer^22^ (version 1.0.5; default settings), was used to determine Phenolyzer scores for all genes relevant to clinical phenotype based on disease and/or on HPO terms. Phenolyzer uses a range of gene−disease databases (OMIM, Orphanet, ClinVar, Gene Reviews, and GWAS catalogue) in combination with HPO terms to map clinical phenotypes to related diseases and genes. Phenolyzer first identifies a list of diseases that are associated with the input terms and uses the gene−disease databases to link diseases to genes. Each gene receives a weighted sum score (ranging from 0 to 1) corresponding to all reported gene−disease relationships. If a set of HPO terms is associated with the same disease, the genes associated to that disease receive a higher score. A ranked in silico gene list is built using a patient’s disease and HPO terms. Phenoparser was again used to process and store this output in an SQLite database to preserve the results for any future reanalysis.

### Incorporation of an ACMG classifier

To assist in the interpretation of sequence variants, the ACMG and the Association for Molecular Pathology (AMP) developed a set of standards and guidelines^15^ to classify variants as “pathogenic”, “likely pathogenic”, “likely benign”, “benign” or “uncertain significance” based on adherence to a set of evidence-based criteria. Here we focused on assignment to the “pathogenic” or “likely pathogenic” categories with variants not reaching the required evidence being classified as “undefined”. Supplementary Table 1 details the criteria we were able to address and the method by which we did so. To accompany some of the methods (pvs1, pp2), we created a gene:impact:disease database (GIDdb) compiled using data from Ensembl and OMIM that links HGNC gene symbols to OMIM diseases via the sequence ontology impact of the known causal variant.

### Generating web-based clinical reports for ranked variants

The pipeline ranks genes most relevant to a patient’s clinical phenotype and generates a comprehensive, intuitive web-based report (html file) that facilitates viewing and sorting of the ranked list of putative deleterious variants. Variants are ranked according to their presence in genes associated with the patient’s phenotype as determined using the OMIM API and/or by Phenolyzer scores, and then by descending scaled CADD score. That is, a variant that aligns perfectly with a known OMIM disease, or a gene in a phenotypic series, will be ranked highest. Such a variant may also have a perfect Phenolyzer score. Where a match is not obtained by accessing the OMIM API, the variants are ranked by Phenolyzer score alone. Variants are annotated with ACMG pathogenicity criteria and pathogenicity status in ClinVar. ACMG pathogenicity scores are not used in the ranking but are provided to aid the clinical geneticist in reviewing the ranked variants. Specific variants occurring in ≥5 individuals (i.e. numbers of heterozygous individuals plus number of homozygous individuals) across the cohort are highlighted (see below). Of these, those observed ≥6 times are relegated towards the bottom of the ranked list of variants. A column is also included that gives the number of HIGH impact variants seen in the same gene across the cohort dataset. This follows the logic of MacArthur et al.^21^ who found that most genes with three or more independent HIGH impact variants represent systematic sequencing errors. These latter parameters enable the clinician or researcher to evaluate whether a putative causative variant might be due to a common sequencing or alignment error.

### Implementing the bioinformatics pipeline

The pipeline relies on several bioinformatics tools and a configuration file is provided to inform the pipeline where each are installed. The tools and versions used to generate the data presented here are given in Table 5. The entire pipeline, including documentation, is bundled with the distribution of Phenoparser, which is available at https://github.com/TimoLassmann/Phenoparser. A simple shell script is provided to run each step of the pipeline, which can be modified for use within workflow management software such as Bpipe^51^ to further maintain reproducibility.

**Table 5:** Software components of the pipeline.

Software	Description of use in this pipeline	Version	Availability
vt	Decomposition and Normalization of Variants	v0.57721	https://genome.sph.umich.edu/wiki/Vt
Variant Effect Predictor (VEP)	Variant Annotation	v84	http://www.ensembl.org/info/docs/tools/vep/script/index.html
GEMINI	Storage of annotated variants	0.30.1	https://github.com/arq5x/gemini
OMIM API	Retrieval and linkage of OMIM terms to causative genes	As accessed on 28/06/19	https://www.omim.org/api
Phenolyzer	Linkage of HPO terms to causative genes	v1.0.5	http://phenolyzer.wglab.org
Phenoparser	Process and storage of OMIM and Phenolyzer results and generation of gene panels	v1.0.0	https://github.com/TimoLassmann/Phenoparser
BCFtools	Manipulation of variant data files	v1.6	http://www.htslib.org/download
HTSlib (includes tabix and bgzip)	Indexing variant data files	v1.6	http://www.htslib.org/download
Grabix	Indexing variant data files	0.1.8	https://github.com/arq5x/grabix
R	Collation of data and generation of patient report files	3.2.3	https://www.r-project.org

### Other pipelines

We compared rankings that were achieved in our pipeline for previously diagnosed cases with those obtained using Exomiser^18^ and AMELIE^19^, performed according to author guidelines. Exomiser input consisted of patient VCF files, whereas rankings were obtained from the AMELIE gene list API using the same genes contained in each SeqNextGen html report as input.

### Reporting summary

Further information on research design is available in the Nature Research Reporting Summary linked to this article.

## Supplementary information

Supplementary Information

Supplementary Data 1

Reporting Summary

## Data Availability

The primary purpose of this paper is to share the computational pipeline developed to analyse patient sequence data. Ethical approval for the study allowed for time-limited sharing of de-identified exome sequencing data from the diagnostic laboratory to the research team. Consent did not include making individual de-identified clinical sequence data available for public access. The clinical sequence data are held under patient confidentiality by the diagnostic laboratory. Downstream patient reports that can be viewed in a web browser are available at https://richardwfrancis.github.io/sng_reports/.

## Code availability

Full open access code is available at https://github.com/TimoLassmann/Phenoparser. All details of software and versions used are provided in Table 5. Any updates to this will be posted on the github site, where queries can also be posted.
