# Supplementary material for: A flexible computational pipeline for research analyses of unsolved clinical exome cases
Source: NPJ Genom Med. 2020 Dec 10;5:54. doi: 10.1038/s41525-020-00161-w (PMC7730424; doi:10.1038/s41525-020-00161-w)
Supplement: Supplementary file 1 — Supplementary Information [file 41525_2020_161_MOESM1_ESM.pdf]

npg Genomic Medicine

ARTICLE

A flexible computational pipeline for research analyses of unsolved clinical exome cases

Timo Lassmann[1,†], Richard W. Francis[1,†], Alexia Weeks[1], Dave Tang[1], Sarra E. Jamieson[1], Stephanie Broley[2], Hugh J. S. Dawkins[3], Lauren Dreyer[2], Jack Goldblatt[2], Tudor Groza[1,2], Benjamin Kamien[2], Cathy Kiraly-Borri[2], Fiona McKenzie[2,4], Lesley Murphy[5], Nicholas Pachter[2], Gargi Pathak[2], Cathryn Poulton[2], Amanda Samanke[6], Rachel Skoss[1], Jennie Slee[2], Sharron Townshend[2], Michelle Ward[2], Gareth S. Baynam[1, 2,4,7], Jenefer M. Blackwell[1] † Equal contributions

[1]Telethon Kids Institute, University of Western Australia, Perth, WA, Australia; [2]Genetic Services of Western Australia, Department of Health, Government of Western Australia, Perth, WA, Australia; [3]Office of Population Health Genomics, Public Health Division, Department of Health, Government of Western Australia, Perth, WA, Australia; [4]Faculty of Health and Medical Sciences, Division of Pediatrics, University of Western Australia, Perth, WA, Australia; [5]Rare Voices Australia, Sydney, Australia; [6]GaRDN Genetics and Rare Diseases Network, WA, Australia; [7]Western Australian Register of Developmental Anomalies, Department of Health, Government of Western Australia, Perth, WA, Australia

Correspondence: Timo Lassmann (Timo.Lassmann@telethonkids.org.au) or Jenefer M. Blackwell (Jenefer.Blackwell@telethonkids.org.au); Telephone: +61 (8) 63191550

SUPPLEMENTARY INFORMATION

Legend to Supplementary Data 1.

Supplementary Data 1. Comparison of parameters influencing pipeline performance for prior compared to candidate and unresolved diagnoses. Bold highlights the previously diagnosed cases that failed in the pipeline. There were 3 prior diagnosis (ASPM, SLC26A4 and SARDH) that were for autosomal recessive disorders where the patient was diagnosed by the diagnostic laboratory as compound heterozygote (note: the second variant for ASPM did not filter through our pipeline). n/k = not known. Het = heterozygous; Hom = homozygous; Hem = male X-chr hemizygous. *,**,***,**** indicate same variants in genes specified.

Supplementary Table 1 Criteria used to assign pathogenicity criteria to variants following ACMG guidelines

Code	Criteria for code assignment	Caution Code	Criteria for assigning caution code
pvs1	null variant in a gene where LOF is a known mechanism of disease	pvs1_caution	Is the variant in the last exon?
ps1	Same amino acid change as a previously established pathogenic variant regardless of nucleotide change	ps1_caution	If ps1 = 1, shows codon change
pm1	Located in a mutational hot spot and/or critical and well-established functional domain without benign variation	pm1_caution	Always empty
pm2	Absent from controls (or at extremely low frequency if recessive)	pm2_caution	Always empty
pm4	Protein length changes as a result of in-frame deletions/insertions in a nonrepeat region or stop-loss variants	pm4_caution	Always empty
pm5	Novel missense change at an amino acid residue where a different missense change determined to be pathogenic has been seen before	pm5_caution	If pm5 = 1, shows known allele change
pp2	Missense variant in a gene that has a low rate of benign missense variation and in which missense variants are a common mechanism of disease	pp2_caution	Shows connection errors if any
pp3	Multiple lines of computational evidence support a deleterious effect on the gene or gene product	pp3_caution	Shows number of computational tools (n=3) displaying significant scores

Supplementary Figure 1

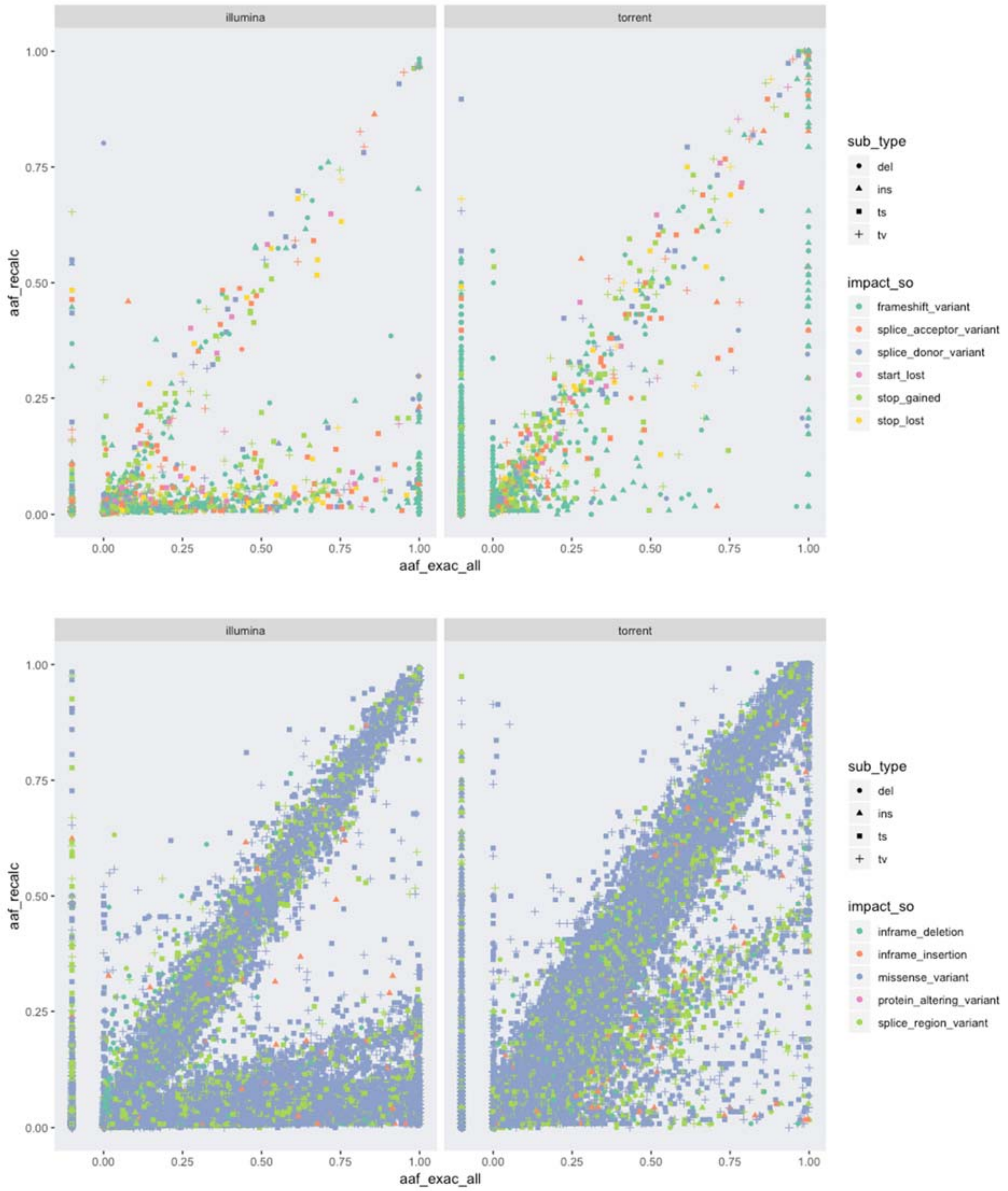

Supplementary Figure 1 Variant annotations by sequencing technology. Cohort allele frequencies (Y axes) by EXAC_all (using the aaf_exac_all variable from GEMINI) frequencies for (a) HIGH impact and (b) MEDIUM impact variants called in Ion Torrent compared to Illumina Trusight™ sequencing. The tower of variants observed with allele frequency of zero, or an apparent allele frequency of one, in EXaC_all (X-axis) compared to the study cohort (Y-axis) are false-positives consistent with systematic alignment errors particularly in Ion Torrent data. These error rates are higher for HIGH impact than MEDIUM impact missense variants.
