# Supplementary material for: A flexible computational pipeline for research analyses of unsolved clinical exome cases
Source: NPJ Genom Med. 2020 Dec 10;5:54. doi: 10.1038/s41525-020-00161-w (PMC7730424; doi:10.1038/s41525-020-00161-w)
Supplement: Supplementary file 3 — Reporting Summary [file 41525_2020_161_MOESM3_ESM.pdf]

nature research

Corresponding author(s): Jenefer M. Blackwell

Last updated by author(s): Sep 2, 2020

Reporting Summary

Nature Research wishes to improve the reproducibility of the work that we publish. This form provides structure for consistency and transparency in reporting. For further information on Nature Research policies, see our Editorial Policies and the Editorial Policy Checklist.

Statistics

For all statistical analyses, confirm that the following items are present in the figure legend, table legend, main text, or Methods section.

n/a	Confirmed	
☐	☒	The exact sample size (*n*) for each experimental group/condition, given as a discrete number and unit of measurement
☒	☐	A statement on whether measurements were taken from distinct samples or whether the same sample was measured repeatedly
☒	☐	The statistical test(s) used AND whether they are one- or two-sided<br>*Only common tests should be described solely by name; describe more complex techniques in the Methods section.*
☒	☐	A description of all covariates tested
☒	☐	A description of any assumptions or corrections, such as tests of normality and adjustment for multiple comparisons
☒	☐	A full description of the statistical parameters including central tendency (e.g. means) or other basic estimates (e.g. regression coefficient) AND variation (e.g. standard deviation) or associated estimates of uncertainty (e.g. confidence intervals)
☒	☐	For null hypothesis testing, the test statistic (e.g. *F*, *t*, *r*) with confidence intervals, effect sizes, degrees of freedom and *P* value noted<br>*Give P values as exact values whenever suitable.*
☒	☐	For Bayesian analysis, information on the choice of priors and Markov chain Monte Carlo settings
☒	☐	For hierarchical and complex designs, identification of the appropriate level for tests and full reporting of outcomes
☒	☐	Estimates of effect sizes (e.g. Cohen's *d*, Pearson's *r*), indicating how they were calculated

Our web collection on statistics for biologists contains articles on many of the points above.

Software and code

Policy information about availability of computer code

Data collection	The TargetSeq Exome V2 kit was used for exome enrichment for sequencing on the SOLiD system. The Ion AmpliSeq Exome RDY kit was used for exome enrichment for sequencing on the Ion Proton System. Illumina ES analysis was carried out using TruSight™ One (~4800 genes) or TruSight™ One Expanded (~6700 genes) panels sequenced on the MiSeq or NextSeq 550 systems.
Data analysis	SOLiD exome sequence data was analysed under LifeScope 2.5. Ion Torrent ES data was analyzed under Torrent Suite 4.2. Illumina exome seqeunce data were analysed using MiSeq Reporter. For Illumina data, BAM files were processed with GATK 4.0.2.0 and SAMtools 1.7. Variants were decomposed and normalised using vt (version v0.57721). Variants were annotated using the Ensembl Variant Effect Predictor (VEP) version 84. Annotated variants were imported into GEMINI47 (version 0.30.1). Phenolyzer (version 1.0.5; default settings), was used. The entire pipeline, including documentation, is bundled with the distribution of Phenoparser, which is available at https://github.com/TimoLassmann/Phenoparser. A simple shell script is provided to run each step of the pipeline, which can be modified for use within workflow management software such as Bpipe to further maintain reproducibility.

For manuscripts utilizing custom algorithms or software that are central to the research but not yet described in published literature, software must be made available to editors and reviewers. We strongly encourage code deposition in a community repository (e.g. GitHub). See the Nature Research guidelines for submitting code & software for further information.

Data

Policy information about availability of data

All manuscripts must include a data availability statement. This statement should provide the following information, where applicable:

- Accession codes, unique identifiers, or web links for publicly available datasets
- A list of figures that have associated raw data
- A description of any restrictions on data availability

Participants gave consent for review of de-identified sequence data but not for these data to be made available in any public domain database as this would be unethical.

Field-specific reporting

Please select the one below that is the best fit for your research. If you are not sure, read the appropriate sections before making your selection.

☒ Life sciences ☐ Behavioural & social sciences ☐ Ecological, evolutionary & environmental sciences

For a reference copy of the document with all sections, see nature.com/documents/nr-reporting-summary-flat.pdf

Life sciences study design

All studies must disclose on these points even when the disclosure is negative.

Sample size	N=179 consented individuals with data suitable for analysis in our pipeline; N=34 with a previous molecular diagnosis; N= 145 with not previous diagnosis.
Data exclusions	No exclusions.
Replication	Not required, the study is designed to make molecular diagnoses of rare diseases on a case-by-case basis.
Randomization	Not required, the study is designed to make molecular diagnoses of rare diseases on a case-by-case basis.
Blinding	Investigators were blind to the previous diagnosis for N=34 cases, which were used as a gold standard to test the ability of the computational pipeline to make a diagnosis.

Reporting for specific materials, systems and methods

We require information from authors about some types of materials, experimental systems and methods used in many studies. Here, indicate whether each material, system or method listed is relevant to your study. If you are not sure if a list item applies to your research, read the appropriate section before selecting a response.

Materials & experimental systems

n/a	Involved in the study	
☒	☐	Antibodies
☒	☐	Eukaryotic cell lines
☒	☐	Palaeontology and archaeology
☒	☐	Animals and other organisms
☐	☒	Human research participants
☐	☒	Clinical data
☒	☐	Dual use research of concern

Methods

n/a	Involved in the study	
☒	☐	ChIP-seq
☒	☐	Flow cytometry
☒	☐	MRI-based neuroimaging

Human research participants

Policy information about studies involving human research participants

Population characteristics	The mean±SD age of participants at time of enrolment was 8.03±6.27 years, median age of 6.83, range 0 to 47 years. All participants were diagnosed with rare diseases.
Recruitment	Participants were recruited through a genetic counsellor at Genetic Services of Western Australia (GSWA), King Edward Memorial Hospital, Perth, Australia. All individuals were engaged through the rare and undiagnosed diseases diagnostic service (RUDDS). Participants, or their carers (for participants aged <18 years of age or >18 years with reduced capacity to consent), gave written consent to share their de-identified ES or targeted NGS-sequenced exome data and relevant clinical phenotypic information with the SeqNextGen study. Only a clinical geneticist at Genetic Services of Western Australia had the authority to re-identify a participant to provide feedback of genetic results as they pertained to the rare disease

diagnosis. Feedback is only provided for fully validated variants. Secondary findings (i.e. information on genetic variants not related to the individual's primary rare disease phenotype) were not gathered or reported. Participants were eligible to take part in the SeqNextGen study if they had given prior clinical consent for genetic diagnosis of their rare disease using exome sequencing.

Ethics oversight	Ethical approval for the study (known as SeqNextGen) was obtained from the Human Research Ethics Committee at Princess Margaret Hospital for Children, Perth, Australia (#2105034EP) and the Department of Health Research Governance Service (#RGS2494).

Note that full information on the approval of the study protocol must also be provided in the manuscript.

Clinical data

Policy information about clinical studies

All manuscripts should comply with the ICMJE guidelines for publication of clinical research and a completed CONSORT checklist must be included with all submissions.

Clinical trial registration	This was not a clinical trial
Study protocol	see above
Data collection	see recruitment above, but note this was not a clinical trial
Outcomes	outcomes were on an individual basis for patients with rare genetic diseases; see notes above on restrictions on clinical feedback
